# From theory to experiments for testing the proximate mechanisms of mast seeding: an agenda for an experimental ecology

**DOI:** 10.1111/ele.13442

**Published:** 2019-12-19

**Authors:** Michał Bogdziewicz, Davide Ascoli, Andrew Hacket‐Pain, Walter D. Koenig, Ian Pearse, Mario Pesendorfer, Akiko Satake, Peter Thomas, Giorgio Vacchiano, Thomas Wohlgemuth, Andrew Tanentzap

**Affiliations:** ^1^ Department of Systematic Zoology Faculty of Biology Adam Mickiewicz University in Poznań Umutlowska 89 61‐614 Poznań Poland; ^2^ Department of Agricultural, Forest and Food Sciences University of Turin 10095 Grugliasco Torino Italy; ^3^ Department of Geography and Planning School of Environmental Sciences University of Liverpool Liverpool UK; ^4^ Lab of Ornithology Cornell University Ithaca NY 14850 USA; ^5^ Fort Collins Science Center U.S. Geological Survey Fort Collins CO USA; ^6^ Institute of Forest Ecology Department of Forest and Soil Sciences University of Natural Resources and Life Sciences Vienna Austria; ^7^ Department of Biology Faculty of Science Kyushu University 819‐0395 Fukuoka Japan; ^8^ School of Life Sciences Keele University Staffordshire ST5 5BG UK; ^9^ DISAA University of Milan via Celoria 2 20123 Milano Italy; ^10^ Swiss Federal Institute for Forest, Snow and Landscape Research WSL Forest Dynamics, Zürcherstrasse 111 CH‐8903 Birmensdorf Switzerland; ^11^ Department of Plant Sciences University of Cambridge Downing St Cambridge CB2 3EA UK

**Keywords:** experimental framework, mast seeding, masting, plant reproduction, research agenda

## Abstract

Highly variable and synchronised production of seeds by plant populations, known as masting, is implicated in many important ecological processes, but how it arises remains poorly understood. The lack of experimental studies prevents underlying mechanisms from being explicitly tested, and thereby precludes meaningful predictions on the consequences of changing environments for plant reproductive patterns and global vegetation dynamics. Here we review the most relevant proximate drivers of masting and outline a research agenda that takes the biology of masting from a largely observational field of ecology to one rooted in mechanistic understanding. We divide the experimental framework into three main processes: resource dynamics, pollen limitation and genetic and hormonal regulation, and illustrate how specific predictions about proximate mechanisms can be tested, highlighting the few successful experiments as examples. We envision that the experiments we outline will deliver new insights into how and why masting patterns might respond to a changing environment.

## Graphical abstract

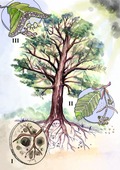

## Introduction

Masting, or mast seeding, the highly variable and synchronised seed production by plant populations (Kelly 1994; Crone & Rapp 2014), is a widespread reproductive strategy in perennial plants (Kelly & Sork 2002; Tanentzap & Monks 2018; Fernández‐Martínez *et al.* 2019). The resulting resource pulses have cascading effects on plant and animal population dynamics, macronutrient cycling and disease risk in humans (Ostfeld & Keesing 2000; Bogdziewicz *et al.* 2016; Vacchiano et al. 2018). From an evolutionary perspective, masting results from economies of scale, that is, individual plants that reproduce when other plants are also flowering or seeding have lower costs per surviving offspring (Kelly 1994). The two most supported selective pressures for economies of scale are predator satiation in years with large seeds crops, which enhance seed and seedling survival, and increased pollination efficiency in high‐flowering years (Kelly & Sork 2002; Pearse et al. 2016).

On a proximate level, masting emerges by combining two processes: annual variability in seed production and synchronisation among individuals (Herrera 1998; Koenig *et al.* 2003). Several hypotheses have been proposed to explain the proximate drivers of masting, but it remains unclear to what extent these are valid or how they are conserved among or even within species (Kelly *et al.* 2013; Crone & Rapp 2014; Pearse *et al.* 2014; Monks *et al.* 2016). Observational studies of masting patterns amassed over the past 50 years have led to considerable theoretical advances, yet there have been few experimental tests of those theories (Crone *et al.* 2009; Smaill *et al.* 2011; Pearse et al. 2015).

Global synthesis of plant reproductive patterns shows that seed production has declined and become more variable over the last 100 years (Pearse et al. 2017). Yet, we have little idea what has driven this change. Prior studies have predicted that masting intensity will increase, decrease, or remain unchanged in response to climate change (Kelly *et al.* 2013; Koenig *et al.* 2015; Monks *et al.* 2016; Bogdziewicz *et al.* 2017b). This uncertainty may partly arise from the fundamentally different mechanisms that appear to underlie masting in closely related taxa (Table 1) (Koenig *et al.* 2016; Pearse et al. 2016; Bogdziewicz *et al.* 2017c). Experiments are now needed both to understand the mechanisms underlying masting, and to better predict the consequences of a changing climate for plant reproductive patterns and global vegetation dynamics.

**Table 1:** Summary of selected observational studies supporting different proximate mechanisms of masting seeding in commonly studied taxa

Taxa	Resource dynamics			Pollination dynamics			Genetic and hormonal regulation
	*Hypotheses*						
	Matching	Switching	Storage	Pollen coupling	Phenological synchrony	Aerial diffusion	
*Quercus*	+ *Q. ilex* 1	+ *Q. lobata, Q. douglasii*, *Q.agrifolia* 2	+ *Q. petraea*, *Q. robur* 3, *Q. rubra*, *Q. alba* 4, *Q. lobata* 5	+ *Q. douglasii* *6*	+ *Q. lobata* 7, *Q. petraea*, *Q. robur* 8 *, Q. ilex* 9	+ *Q. petraea*, *Q. robur* 3	Not studied
*Fagus* & *Nothofagus*	No evidence^11^, ^12^	+ *F. sylvatica* 13, *N. truncata* 11	+ *F. crenata* 12	+ *F. sylvatica* 14 *, F. crenata* 15, *N. solandri*, *N. menziesii* 16	No evidence^8^	Not studied	Combination of genetic and environmental signals regulate flowering gene expression in *F. crenata* 12, 17
*Chionochloa*	No evidence^18^	+ 5 *Chionochloa* species^19^	+ 5 *Chionochloa* species^18^, ^20^	*Chionochloa* are self‐compatible, so pollination is not expected to be important role in synchronizing their reproduction^18^			High temperature‐induced increases in gibberellin levels promote flowering^21^

+: Supported; ‐: Not supported.

^1^ Pérez‐Ramos et al. (2010);

^2^ Barringer et al. (2013);

^3^ Schermer et al. (2019);

^4^ Bogdziewicz et al. (2018);

^5^ Pesendorfer et al. (2016);

^6^ Knapp et al. (2001);

^7^ Koenig et al. (2015);

^8^ Bogdziewicz et al. (2017c);

^9^ Bogdziewicz et al. (2017b);

^11^ Monks & Kelly (2006);

^12^ Abe et al. (2016);

^13^ Hacket‐Pain et al. (2018);

^14^ Nilsson & Wastljung (1987);

^15^ Kon et al. (2005);

^16^ Kelly et al. (2001);

^17^ Satake et al. (2019b);

^18^ Rees et al. (2002);

^19^ Tanentzap et al. (2014);

^20^ Monks et al. (2016);

^21^ Turnbull *et al. * (2011).

Our aim here is to outline a research agenda that takes the biology of masting from a largely observational field of ecology to one rooted in mechanistic understanding. This understanding can be incorporated into global vegetation models to improve their accuracy and realism in terms of seed production but also growth tradeoffs, seed dispersal, establishment, migration, cascading trophic interactions, and ecosystem resilience to disturbances or climate change (Vacchiano et al. 2018; Clark *et al.* 2019). We outline explicit predictions of prevalent hypotheses explaining intermittent and synchronised reproduction at the population level and describe what experiments would be necessary to test them. We do not try to repeat previous reviews of masting theory (Crone & Rapp 2014; Pearse et al. 2016; Allen *et al.* 2018; Vacchiano et al. 2018). Rather, we illustrate how specific predictions about the proximate mechanisms involved in masting can be tested and highlight successful experiments as examples.

## Hypotheses, predictions and experimental tests

**Figure 1:**
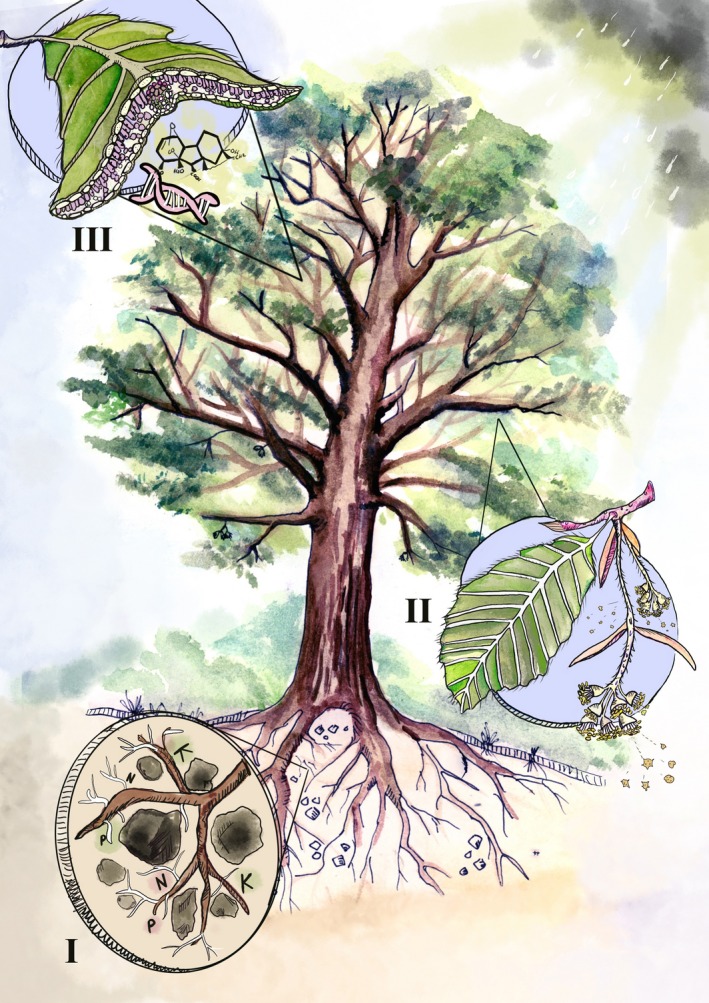
Main processes responsible for driving mast seeding: resource dynamics (I), pollination (II), hormonal and genetic expression (III), all of which are influenced by environmental variation. To produce a mast crop, plants in a population need to initiate many flowers, these flowers need to be pollinated at a high rate, and fertilised flowers need to mature into seeds. The mechanisms responsible for masting determine the success of transition from one seed developmental phase to another and thus population‐wide synchrony.

We divide our discussion into the three main processes underpinning mast seeding: resource dynamics, pollen limitation and genetic and hormonal regulation (Fig. 1). Environmental variation has been traditionally recognised as a masting driver, but its effect is largely, if not exclusively, through these processes. Thus, the discussion of environmental variation as a masting driver is incorporated into the three aforementioned sections.

### Resource dynamics

#### Theoretical predictions

**Figure 2:**
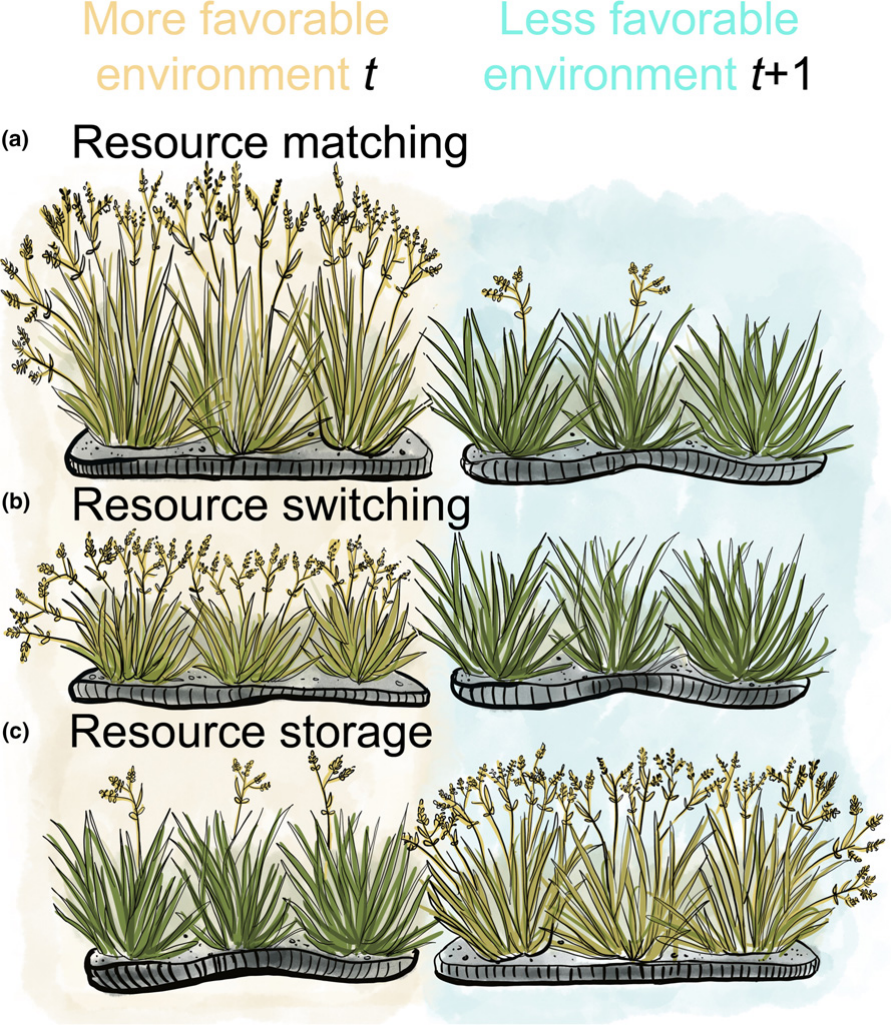
Graphical representation of resource matching, switching and storage hypotheses. Left‐hand panel shows plants in environmentally favourable years, whereas right‐hand panel shows plants in the following and less‐favourable year. (a) Resource matching predicts that environmentally favourable years should result in both higher growth and reproduction. (b) Resource switching predicts that environmentally favourable years result greater investment in reproduction at the cost of growth. (c) Resource storage predicts large reproductive investment once plant accumulates enough resources.

The internal resource dynamics of individual plants are potentially responsible for annual variation in individual seed production in at least three ways (Fig. 2) (Pearse et al. 2016). The first two hypotheses predict that resources are allocated for either reproduction or growth within each year, whereas the third hypothesis predicts that resources are carried over between years. First, the resource matching hypothesis predicts that a fixed fraction of resources is allocated to reproduction each year. Annual variation in seed production is thus a consequence of annual variation in resource acquisition. Resource matching is essentially a null hypothesis for mast seeding, wherein annual variability in seed production entails no adaptive framework beyond using what resources are available each year for reproduction.

There are at least two adaptive alternatives to resource matching. One is the resource switching hypothesis, which predicts that a variable fraction of current‐year resource acquisition is allocated to seed production (Monks & Kelly 2006; Hacket‐Pain *et al.* 2018). Years with more available resources see greater investment in reproduction, whereas years with fewer available resources result in more investment in plant growth and less reproduction. Thus, the ratio between vegetative to reproductive allocation should vary with resource switching but remain constant under resource matching.

Finally, the resource storage hypothesis predicts that plants accumulate resources over several years, eventually investing them in a large ‘mast crop’ (Isagi *et al.* 1997; Satake & Iwasa 2000; Han *et al.* 2014 ). Storage can be active if plants store resources until a certain resource threshold is reached, or passive if environmental constraints limit seed production in some years, forcing plants to save resources for reproduction in subsequent years (Pesendorfer *et al.* 2016; Bogdziewicz *et al.* 2018).

#### Experimental tests

The most obvious way to test how resources are involved in seed production is to supplement different macronutrients nitrogen, phosphorous, carbon – at different seed developmental phases. Ideally, this experiment would be replicated across different species, and flower initiation, anthesis and seed maturation would be monitored to differentiate between ‘flowering masting’ and ‘fruit maturation’ species in which annual variability in seeding is primarily driven by differences in flower production and fruit abortion, respectively (Pearse et al. 2016). Under resource matching, the addition of resources should increase both current growth and reproduction, whereas resource switching predicts disproportionate investment in current reproduction. In contrast, the addition of resources beneath a threshold required to induce flowering would increase seed production only in later years if resource storage were important. In the absence of *a priori* knowledge about this threshold, resources would need to be added at different levels.

Resource addition experiments have thus far yielded variable results. A likely explanation for this variability is the potential for different macronutrients to be limiting in different species and both the differing time scales and phenological stages at which resources matter (Miyazaki *et al.* 2014; Pulido *et al.* 2014; Minor & Kobe 2017; Bogdziewicz *et al.* 2017a; Brooke *et al.* 2019). Such differences highlight the desirability of performing fully factorial experiments on a variety of masting species over multiple years.

An excellent example of a resource addition experiment is that of Smaill *et al. *(2011), who investigated the effect of N fertilizer in *Nothofagus solandri* stands. They found that fertilization increased seed production, but only in some years. This variability was attributed to different responses to weather depending on the treatment. Seed production in unfertilized stands was primarily linked to rainfall the year before dispersal (higher rainfall leading to greater N mineralization and uptake), while in fertilized stands where N limitation was removed, seed production was affected mainly by temperature during flower primordia development. Analogous results were obtained by Miyazaki *et al. *(2014), who combined N fertilization with monitoring of flowering gene expression levels in *Fagus crenata* and found that N addition stimulated flower transition and mass flowering in consecutive years. These studies demonstrate the key role and interaction of resources and environmental variation in driving masting, but they do not explicitly test the resource‐related hypotheses outlined above.

A second experimental approach is to prevent seed maturation, typically the most resource‐demanding phase, by harvesting seeds before they ripen or applying ethylene inhibitors designed to reduce or eliminate flowering (Bukovac *et al.* 2006). This treatment should result in larger seed production in the next year only under the resource storage hypothesis, but would not differentiate resource matching from switching. Results that are more conclusive are likely to be generated by experiments that not only prevent seed maturation but, conversely, encourage plants to produce more seeds. These outcomes can be achieved with agricultural sprays that inhibit biosynthesis of ethylene, thereby forcing plants to retain flowers that are otherwise likely to be aborted. This approach could prove particularly powerful combined with tracking analyses of potentially key macronutrients.

Thus far the most influential experiment conducted along these lines has been that of Crone *et al. *(2009) studying the wildflower *Astralagus scaphoides*. These authors removed flowers from some plants for one year and from others for three consecutive years to desynchronize flowering. The experiment demonstrated that seed production in this species depletes stored carbohydrates and limits subsequent flowering. Asynchronously flowering plants failed to produce seeds due to density‐dependent pollen limitation, but they did not deplete carbohydrate stores and were able to flower in following years and resynchronize with the rest of the population, supporting the resource storage hypothesis.

Experiments that simulate environmental conditions projected by global change models, like warming temperatures, CO_2_ fertilization, or rainfall exclusion, are also useful for understanding the impacts of resource dynamics on the reproductive patterns of masting plants (LaDeau & Clark 2001; Chung *et al.* 2013; Pérez‐Ramos *et al.* 2013; Bykova *et al.* 2018). The effects of these treatments will depend on how resource dynamics initially influence masting. In the case of water limitation experiments, drought interacts with the acquisition and storage of other resources (Pearse et al. 2016), in addition to potentially serving as the environmental cue synchronizing reproduction within populations (Espelta et al. 2008, see also section IIIa). In the latter case, reproduction of masting species that use water shortage as a cue should be strongly affected by water limitation, as selection would favour plants that are sensitive to drought to foster synchrony (Bogdziewicz *et al.* 2019b).

Potential complications in experimental tests of resource dynamics, and in all masting experiments more generally, may arise if species take multiple years to develop their seeds (Knops *et al.* 2007). Furthermore, resources added to plants or carried forward to the next year may not be immediately invested into seeds due to poor weather conditions, such as frost or a lack of weather cues required to initiate flowering (Rees *et al.* 2002; Abe *et al.* 2016; Monks *et al.* 2016; Bogdziewicz *et al.* 2018). Thus, cohorts of control and experimental plants must be observed for several years so that differences in environmental conditions can be considered.

### Pollen limitation

#### Theoretical predictions

Even if endogenous resource dynamics induce the observed annual variability at the individual level, plants require a synchronizing factor to produce population‐wide mast seeding. Recent work supports the hypothesis that pollen limitation – up until recently a factor whose role in masting was unclear (Koenig & Ashley 2003), particularly in wind pollinated species (Koenig *et al.* 2012; Pearse et al. 2015) – can be that synchronizing factor.

**Figure 3:**
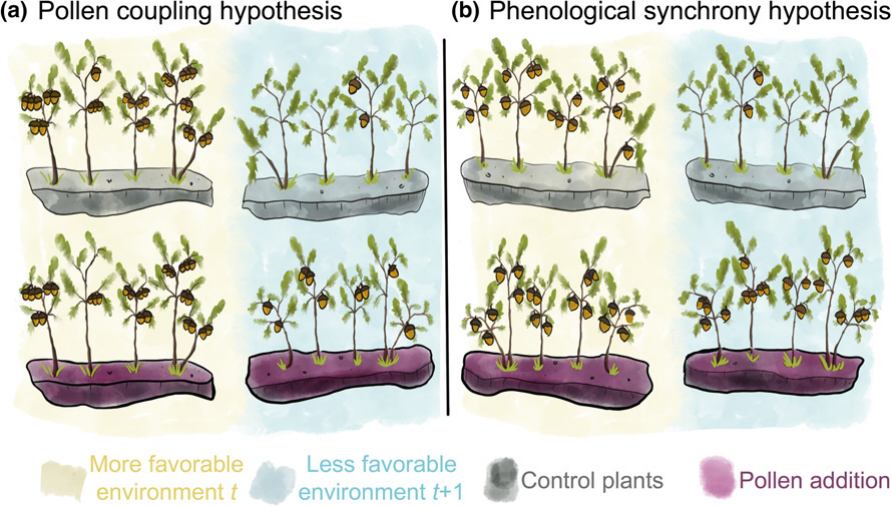
Graphical representation of pollen coupling and phenological synchrony hypotheses. Under the (a) pollen coupling hypothesis, the low density of flowering results in pollen limitation irrespective of environmental favourability. Under the (b) phenological synchrony hypothesis, pollen limitation may also happen in years when flowering density is high but the within‐year synchrony of flowering is low. Top panels show control plants, while plants in bottom panel receive pollen‐addition treatments.

Pollen limitation may drive synchronization of seed production in several non‐exclusive ways (Fig. 3). The first is density‐dependent pollen coupling, which predicts that annual variation in density of flowering plants drives pollen limitation in self‐incompatible plants (Satake & Iwasa 2000; Kelly *et al.* 2001; Venner et al. 2016). In combination with the resource storage hypothesis, pollen coupling predicts that if a plant flowers out of synchrony with its neighbours, it will not receive pollen, will fail to fertilize flowers, will not deplete resources, and will thus flower again in subsequent seasons until other plants in the population flower. When this last step finally happens, flowers will be pollinated and mature into fruits, which will deplete resources and synchronize the endogenous resource dynamics of the individual with the rest of the population.

Pollen coupling focuses on among‐year variation in flowering synchrony and potentially confers a functional benefit to masting as one of several economies of scale along with, most obviously, predator satiation (Pearse et al. 2016). At the within‐year level, the main mechanism by which pollen limitation is likely to be expressed is phenological synchrony (Koenig *et al.* 2015). Plants that flower in synchrony with a higher number of other individuals experience less pollen limitation. In contrast, low flowering synchrony decreases pollen availability and increases pollination failure. The strength of phenological synchrony is in turn driven by weather. Such population‐wide pollination outcomes may interact with either resource storage or resource switching to produce mast years when large resource pools coincide with high pollination success (Koenig *et al.* 2015; Pesendorfer *et al.* 2016; Bogdziewicz *et al.* 2017b).

There are at least two mechanisms through which weather variability can affect phenological synchrony. The microclimatic hypothesis, proposed originally as a part of the phenological synchrony hypothesis (Koenig *et al.* 2015), predicts that flowering is more asynchronous when microclimatic conditions are more heterogeneous, conditions that translate into greater variability in flowering time. As an example, trees in valleys and at lower elevations are likely to flower later because cold air descends at night, thereby magnifying the microhabitat variation when average temperatures are cooler. Conversely, a relatively homogeneous microclimate in warm years results in synchronous flowering and pollen production and presumably higher pollination success.

An alternative proposed here is the photoperiod sensitivity hypothesis, whereby flowering synchrony can be driven by an interaction between daylength and temperature. In cold years, days are already long when spring warming occurs, reducing the effect of a plant’s daylength sensitivity on its flowering time (Fu *et al.* 2019). In warm years, the days are still short when spring warming occurs, preventing day‐length sensitive plants from flushing and flowering. Thus, in warm years, leaf‐out and flowering advance in day length‐insensitive individuals, but not in day length‐sensitive individuals. Although we know of no explicit tests of this hypothesis, experiments have confirmed large intraspecific variation in day‐length sensitivity within populations of some species (Zohner *et al.* 2018). Consequently, this response may increase the population‐level variability of flowering synchrony under short day conditions (warm years, early spring) and increase synchrony of flowering in late springs (cold years, late spring).

Another hypothesis relating weather and pollen limitation posits that warm, dry temperatures during the pollination period increases pollination efficiency through providing good conditions for pollen release and aerial diffusion (Schermer *et al.* 2019). Thus, this aerial diffusion hypothesis predicts that warm temperatures and dry conditions should decrease pollen limitation through enhancing aerial pollen abundance and dispersal. Pollen limitation may also be a consequence of unfavourable weather events like rainfall washing out pollen from the air column (García‐Mozo *et al.* 2007). As in the case of phenological synchrony, such population‐wide pollination outcomes may interact with resource dynamics to produce mast years (Schermer *et al.* 2019).

#### Experimental tests

Pollen limitation can be tested by pollen addition experiments. Additions conducted along a density gradient of flowering plants either in time (in high‐ and low‐flowering years) or in space would test the strength of pollen coupling, which predicts that the positive effect of pollen addition on seed set should be negatively related to the density of flowering plants. The phenological synchrony hypothesis can be examined by combining pollen additions with monitoring of flowering times, the prediction being that the effect of pollen addition should be stronger in individuals whose phenology is less synchronized with other plants in the population.

There have been few attempts to manipulate pollen levels experimentally, at least in the wind‐pollinated species that disproportionately exhibit masting. In the case of phenological synchrony, no experimental test has been conducted. Similarly, pollen coupling has been tested only in one system. Crone & Lesica (2006) added pollen to flowers of mast‐seeding *A. scaphoides* and found increased seed set in years when a low proportion of the population flowered, but no effect in years when the density of flowering plants was high. This result confirmed the density‐dependence of pollination success in this insect‐pollinated species. Pearse et al. (2015) also added pollen to wind‐pollinated California valley oak (*Quercus lobata*), but without explicitly exploring whether pollination success was determined by pollen coupling or phenological synchrony. They found increased seed set in one of two years, suggesting that interannual variability in pollen limitation synchronizes seed set consistent with models of mast seeding. Their study also demonstrated that most female flowers were aborted due to factors other than a lack of pollination, leaving considerable remaining uncertainty about the proximate mechanisms involved in masting in this species.

A complication of pollen addition experiments is that fruit maturation can be limited by a scarcity of both pollen and resources. Thus, when resources are limiting, supplementing pollen will not result in greater flower‐to‐fruit transitions. Future experimental attempts should try to discriminate these two factors by crossing pollen addition experiments with resource monitoring or supplementation.

Weather can further complicate experimental tests of pollen limitation by influencing flowering. Manipulating among‐plant variation in microclimatic conditions by applying different levels of shading and/or warming can help determine whether microclimatic heterogeneity or the interactive effects of photoperiod and temperature drive flowering synchrony. For example, warm temperatures under short‐day conditions should desynchronize flowering under the photoperiod sensitivity hypothesis, while daylength should be unimportant under the microclimatic hypothesis. Similar setups can be used to test whether higher air temperature around a plant enhances aerial pollen concentrations. No experimental tests of weather variation on pollen limitation have thus far been conducted.

### Genes and hormones

#### Theoretical predictions

To the extent that masting is driven by resources and pollen, plants must have mechanisms to sense their environment and control investment in reproduction as a function of that environment. These mechanisms map onto genetic and hormonal apparatuses that control seed set and are central to understanding the basis of masting (Pearse et al. 2016; Satake *et al.* 2019a,b). Changes in gene expression and resultant changes in hormone secretion can consequently produce both annual variability and synchrony of seed production.

**Figure 4:**
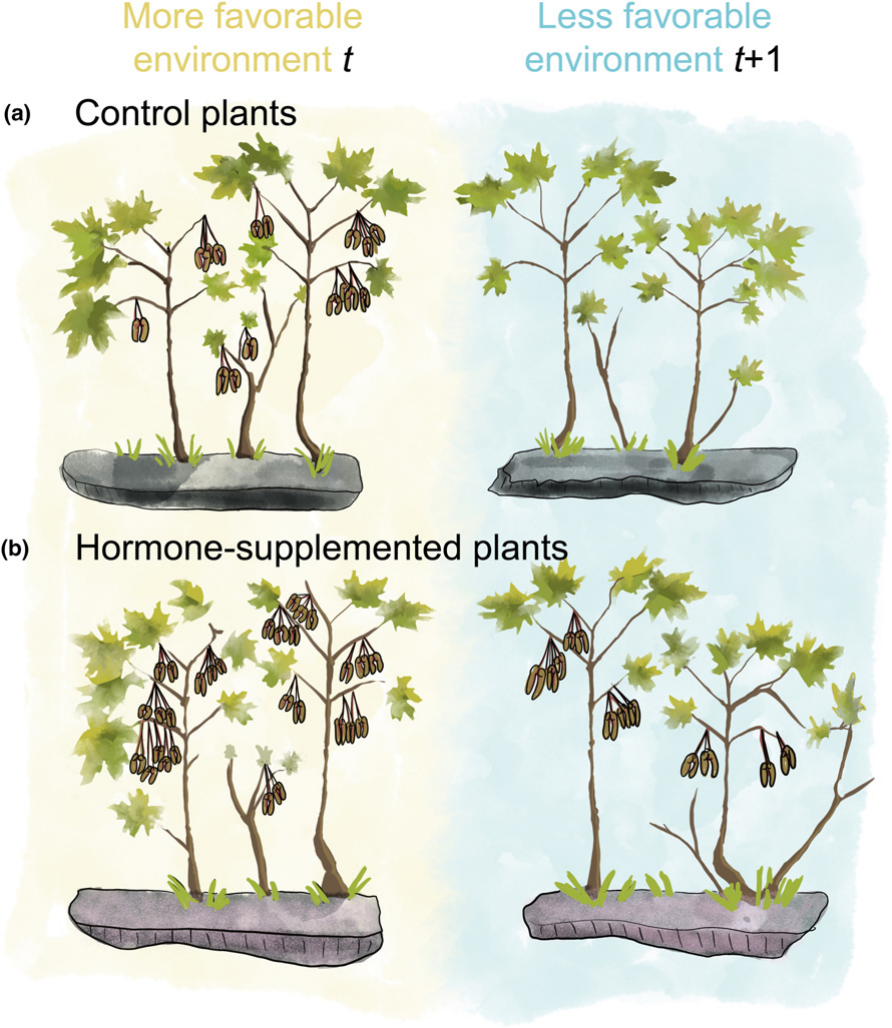
Graphical representation of weather cueing hypothesis. Experiments should monitor plants that are (a) controls (no hormone additions) and (b) supplemented with flowering hormones. Left‐hand panel shows plants in environmentally‐favourable years, whereas right‐hand panel shows plants in the following and less‐favourable years.

Most theory concerning the role of gene expression and associated hormonal secretion in controlling masting has been developed around their interaction with the environment (Pearse et al. 2016). If gene regulatory networks integrate multiple signals such as temperature, nutrients and photoperiod, flowering and fruiting may happen only when all these different signals are received. If these different signals are integrated in an additive manner, a single very strong signal may be sufficient to activate genes for floral transition (Mangan & Alon 2003; Kalir *et al.* 2005). In other words, if hormones and the genes that control them are hypersensitive to an environmental signal, masting can be at least partially independent of resource‐ and pollen‐based mechanisms. The best developed example of this idea is the weather cueing hypothesis (Fig. 4), which predicts that large seasonal deviations from mean weather values trigger changes in flowering gene expression and associated hormone synthesis responsible for initiating bud formation, flower induction, or flower abortion (Kelly *et al.* 2013; Monks *et al.* 2016; Ascoli *et al.* 2017; Vacchiano *et al.* 2017). If regulatory networks are strongly conserved within populations, plants should all respond to the cue in the same way, resulting in high synchrony and among‐year variability in reproduction. There is no requirement for the weather cues to be correlated with higher resource acquisition rates, and the only absolute requirement is that the cue be spatially synchronous over wide areas so all plants can respond similarly (Kelly 1994). The specific link between weather signals and seeding can thus be species‐ and possibly even population‐specific (Bogdziewicz *et al.* 2019a). Nonetheless, the general prediction is that the cue should trigger hormone synthesis and affect flowering in a similar way across individuals within populations.

An untested assumption is that masting behaviour (either synchrony or variability of seed production) is a heritable trait, whereby offspring respond similarly to the environment as their parents (Pearse et al. 2016, Koenig et al. 2017). Evidence for heritability is, however, limited. Only one study has explored this issue in a masting species, *Quercus robur*, finding substantial genetic contributions to variability in masting behaviour (Caignard *et al.* 2019). Assessing heritability based on parental regression (comparing parent and offspring traits) or known siblings (comparing similarity between siblings who share both a biological mother and a father) requires long‐term data on seed production by individual plants of known genetic relatedness, or rearing offspring from known parents in a common environment (Caignard *et al.* 2019). Another way forward may be to correlate the substantial variation in masting traits of individuals (Koenig *et al.* 2003; Crone *et al.* 2011) with their relatedness, but this has yet to be attempted.

#### Experimental tests

Experimental tests of the weather cueing hypothesis require manipulating weather variability to simulate cues identified by previous correlational studies. For instance, if flowering appears to be related to relatively warmer years, an experiment could warm plants to trigger masting events. As an example, Kon & Noda (2007) tested the effect of night‐time temperatures on flower bud initiation in *Fagus crenata* by heating fruit‐bearing branches at different times of flower development. They found that warm temperatures during sensitive development periods vetoed flower initiation and hypothesized that this was because of temperature‐related gibberellin secretion.

Measuring gene expression levels or hormonal levels in vegetative versus reproductive plant organs before, during and after applying the cue will help unravel the mechanisms through which plants perceive cues. As a successful example, field transcriptome analysis using the mass flowering tree *Shorea beccariana* showed that expression levels of drought‐responsive and sucrose‐induced genes increased significantly prior to anthesis (Kobayashi *et al.* 2013). Yeoh *et al. *(2017) applied a molecular phenology approach (Kudoh 2016) to tropical trees in *Shorea* to identify proximate environmental cues for community‐level masting. The activation of flowering genes was observed twice over four years, and was always followed by anthesis. This result was consistent with the occurrence of interacting drought and cool temperature signals (Chen *et al.* 2018). A fully factorial design in which pollen and macronutrients are added *ad libitum* will further test whether, or to what extent, weather acts as a distinct mechanism from pollen limitation and resource dynamics.

An alternative experiment would be to manipulate directly the hormonal cues presumed to be involved in masting without altering resource or pollen availability. One such study exogenously applied two gibberellins (GA3 and GA4) to snow tussocks (*Chionochloa pallens* and *C. rubra*), which increased flowering in some, but not all, years (Turnbull *et al.* 2011). Gibberellin addition appeared to interact with temperature cues correlating with increased flowering. This finding suggested that temperature‐regulated endogenous gibberellin biosynthesis is a causal factor in mast flowering events. In oaks, preliminary studies suggest that manipulating ethylene signaling is critical to explain rates of flower abortion (Pearse et al. unpublished). Because differential flower abortion is the primary cause of interannual variation in oak seed crops (Espelta et al. 2008; Pérez‐Ramos *et al.* 2010; Pearse et al. 2015), ethylene appears to be a strong candidate as a hormonal driver of masting in this taxon.

Examining the molecular basis of environmental cues, such as weather, and testing whether it is resource‐dependent would be a valuable area of future experimentation. A groundbreaking study employing gene expression profiling techniques was that of Miyazaki *et al. *(2014), who monitored expression levels of key flowering‐time genes, *FLOWERING LOCUS T* (*FT*), *LEAFY* (*LFY*) and *APETALA1* (*AP1*) for five years in *Fagus crenata*. *FT* moves from leaves to shoot meristems where it acts to induce flowering, while *LFY* and *AP1* have been identified as necessary for the determination of the floral meristem identity in *A. thaliana* (Mandel & Yanofsky 1995). The expression levels of these flowering genes showed clear between‐year fluctuations in *Fagus crenata* that were associated with a variable flowering and fruiting pattern. Crucially, nitrogen fertilization experiments identified N as a key regulator for the floral transition in this species (Miyazaki *et al.* 2014), showing how resource dynamics maps onto a genetic apparatus that controls seed set.

### Concluding remarks

Despite the crucial role of mast seeding in plant regeneration and many other ecological processes (Ostfeld & Keesing 2000; Schmidt & Ostfeld 2003; Boutin *et al.* 2006; Szymkowiak & Kuczyński 2015; Vacchiano *et al.* 2018), our understanding of its behaviour is mostly based on observational records from natural conditions. Few experiments have been designed to test the predictions of hypotheses for the proximate causes of masting. For example, some of the best experimental tests of resource‐ and pollen‐based hypotheses have come from the bee‐pollinated *Astralagus scaphoides* (Crone *et al.* 2009), but the relevance of these findings to more widespread, wind‐pollinated masting systems, such as long‐lived trees, remains unclear. For weather cueing, experimental tests need to generalise more broadly whether correlations between seeding and weather variation are accompanied by changes in gene expression and associated hormone secretion within a broader regulatory network, or instead reflect mechanisms such as resource or pollen limitation (Pearse *et al.* 2014). Future progress depends on experiments designed to test these hypotheses. As the relative importance of different mechanisms is likely to vary among species, standardized experiments across diverse life strategies would be highly beneficial.

We have summarized potential tests of the mechanisms involved in synchronous and intermittent reproduction (Table 2), thereby outlining ways to improve our understanding of mast seeding. We envision that these experiments will deliver new insights into how and why masting patterns might respond to a changing climate and macronutrient cycles. This knowledge can subsequently be incorporated into broader ecosystem‐scale models to aid predictions of vegetation dynamics and biogeochemical cycles (Vacchiano et al. 2018). For example, current dynamic vegetation models rarely allocate carbon to sexual reproduction, and if so, they assume resource matching (Merganičová *et al.* 2019), which is probably unlikely (Pearse et al. 2016). In agricultural systems, this knowledge may help predict the timing of commercially valuable fruit and nut crops, such as apple, citrus and pistachio (Smith & Samach 2013). Finally, a better understanding of the timing of resource pulses associated with masting can help inform wildlife managers of changes in animal populations and the public about potential health risks such as Lyme disease (Ostfeld *et al.* 2006). As masting underpins many ecological processes that are important to human well‐being, the experimental roadmap we have developed here should ultimately transform our understanding of this phenomenon for the next generation.

**Table 2:** Summary of proximate mechanisms believed to drive mast seeding, the theoretical predictions derived from the main masting hypotheses, and exemplary experiments

Mechanism	Hypothesis	Experiment	Prediction	Practical aspects
1) Resource dynamics	Resource matching	Macronutrient addition	Increase in current growth *and* reproduction	‐ Fully‐crossed addition of different macronutrients ‐Monitoring of all seed developmental phases ‐Cohorts of plants need to be observed over multiple years due to potential poor weather conditions preventing immediate investment of added resources into seeds ‐Environmental control can be in greenhouse and with grafts for larger species such as trees ‐Isotopic labeling can track added nutrients
	Resource switching		Disproportionate increase in current reproduction compared to growth, or vice‐versa	
	Resource storage		Increase in seed production only in subsequent years	
	Resource storage	Prevent seed development	Increase in seed production in subsequent years	As above, but excluding the addition of macronutrients
2) Pollen limitation	Pollen coupling	Pollen addition	Effect size of pollen addition is negatively correlated with density of conspecific flowers	‐Pollen addition across populations differing in flowering density or across individuals differently synchronized within the population ‐ requires crossing pollen addition with resource monitoring or supplementation as fruit set can be limited by both pollen and available resources
	Phenological synchrony		Pollen addition results in larger fruit set in less synchronized individuals, with effect size increasing as density of conspecific flowers declines	
	Microclimatic hypothesis (hypothetical driver of annual variation in phenological synchrony)	Manipulating among‐plant variability in micro‐climate conditions	Larger interindividual heterogeneity in microclimate conditions desynchronizes flowering	‐Applying different levels of shading or warming throughout the population
	Photoperiod sensitivity hypothesis (hypothetical driver of annual variation in phenological synchrony)	Simulating early and late springs	Short daylength and high temperatures desynchronize flowering	‐ simulating early (short days, high temperatures) and late (long days, high temperatures) spring in greenhouse conditions ‐Can use grafts for larger plants
	Aerial diffusion	Manipulating air temperature	Warm air temperature (and low humidity) enhances air pollen concentration	‐Simulating warm spring temperatures in a random subset of plants
3) Hormones and genes	Weather cueing	Manipulating weather variability	Weather cue results in larger hormone secretion/ gene expression and flower/ seed production	‐Manipulation of pre‐identified weather signal ‐requires factorial crossing with resource addition as plant responsiveness to the cue may depend on internal resource state

## Authorship

All authors conceived the idea and formulated the basis of the experimental framework. MB and AT led the writing of the manuscript, and all authors provided critical feedback and helped shape the final text.

## Data Availability

No data were used in the manuscript.
